# The Potential of Mycelium and Culture Broth of *Lignosus rhinocerotis* as Substitutes for the Naturally Occurring Sclerotium with Regard to Antioxidant Capacity, Cytotoxic Effect, and Low-Molecular-Weight Chemical Constituents

**DOI:** 10.1371/journal.pone.0102509

**Published:** 2014-07-23

**Authors:** Beng Fye Lau, Noorlidah Abdullah, Norhaniza Aminudin, Hong Boon Lee, Ken Choy Yap, Vikineswary Sabaratnam

**Affiliations:** 1 Mushroom Research Centre and Institute of Biological Sciences, Faculty of Science, University of Malaya, Kuala Lumpur, Malaysia; 2 University of Malaya Centre for Proteomics Research (UMCPR), Faculty of Medicine, University of Malaya, Kuala Lumpur, Malaysia; 3 Drug Discovery Laboratory, Cancer Research Initiatives Foundation (CARIF), Subang Jaya, Selangor, Malaysia; 4 Department of Pharmacy, Faculty of Medicine, University of Malaya, Kuala Lumpur, Malaysia; 5 Advanced Chemistry Solutions, Kuala Lumpur, Malaysia; Carl-Gustav Carus Technical University-Dresden, Germany

## Abstract

Previous studies on the nutritional and nutraceutical properties of *Lignosus rhinocerotis* focused mainly on the sclerotium; however, the supply of wild sclerotium is limited. In this investigation, the antioxidant capacity and cytotoxic effect of *L. rhinocerotis* cultured under different conditions of liquid fermentation (shaken and static) were compared to the sclerotium produced by solid-substrate fermentation. Aqueous methanol extracts of the mycelium (LR-MH, LR-MT) and culture broth (LR-BH, LR-BT) demonstrated either higher or comparable antioxidant capacities to the sclerotium extract (LR-SC) based on their radical scavenging abilities, reducing properties, metal chelating activities, and inhibitory effects on lipid peroxidation. All extracts exerted low cytotoxicity (IC_50_>200 µg/ml, 72 h) against selected mammalian cell lines. Several low-molecular-weight compounds, including sugars, fatty acids, methyl esters, sterols, amides, amino acids, phenolics, and triterpenoids, were identified using GC-MS and UHPLC-ESI-MS/MS. The presence of proteins (<40 kDa) in the extracts was confirmed by SDS-PAGE and SELDI-TOF-MS. Principal component analysis revealed that the chemical profiles of the mycelial extracts under shaken and static conditions were distinct from those of the sclerotium. Results from bioactivity evaluation and chemical profiling showed that *L. rhinocerotis* from liquid fermentation merits consideration as an alternative source of functional ingredients and potential substitute for the sclerotium.

## Introduction

The different morphological/developmental stages of a mushroom (i.e., the fruiting body, mycelium, and sclerotium) contain bioactive components with health-promoting effects. Only a handful of mushrooms are known to form sclerotia in their life cycles. One representative from the Polyporaceae family is *Lignosus rhinocerotis* [as ‘*rhinocerus*’] (Cooke) Ryvarden (synonym: *Polyporus rhinocerus*), which is located throughout tropical regions. It is also popularly referred to as the “tiger’s milk mushroom” (“*cendawan susu rimau*” in Malay) by the local and indigenous communities in Malaysia. Previous chemical investigations on *L. rhinocerotis* focused mainly on its proximate composition [1] and other nutritional attributes, such as fatty acids, vitamins, minerals, and β-glucans [2]; in particular, the physicochemical and functional properties of the sclerotial dietary fibres have been extensively investigated [3]. Among the bioactive components in *L. rhinocerotis*, the water-soluble, polysaccharide-protein complexes and β-glucans have been thoroughly studied for anti-tumour [4] and immunomodulatory effects [5]. On the other hand, little information on the low-molecular-weight constituents is available even though the use of *L. rhinocerotis* as folk medicine for overall wellness and cancer treatment [6] might be attributed to the presence of secondary metabolites with antioxidative (reduction of oxidative stress) and/or cytotoxic effects against cancer cells.

Wild-growing *L. rhinocerotis* make up the main source of these mushrooms; however, supply is limited due to their rarity [2], [6]. Because of this, attempts have been made to domesticate this highly prized mushroom. Abdullah et al. [7] reported that solid-substrate fermentation of the mycelium on agroresidues yielded the fruiting body and sclerotium. In addition, liquid fermentation for the production of mycelium in bioreactors [8] as well as flasks under shaken [2] and static [9] conditions has been documented. Despite the advantages conferred by liquid fermentation for the production of fungal biomass and metabolites [10], the economic potential of the mycelium and culture broth of *L. rhinocerotis* as sources of nutraceuticals has been overlooked due to continued reliance and emphasis on the naturally occurring sclerotium. This is supported by the fact that previous studies on the mushroom’s bioactivities focused solely on the sclerotium [4]–[5], [11]–[13]. Indeed, the sclerotium is a compact mass of hardened mycelium; however, it is not known if the mycelium can substitute for the sclerotium with respect to bioactivities and chemical constituents. Besides, the chromatographic fingerprints of the extracts of *L. rhinocerotis* from different morphological/developmental stages have not been reported. Consequently, the chemical nature of many bioactive, low-molecular-weight compounds in the extracts remains unidentified [11], [13]. Extensive studies were directed at bioactivity screening and metabolite production, but comparative studies on mushroom mycelia from different culture conditions of liquid fermentation (e.g., shaken and static conditions), which could produce varying amounts of active constituents and affect the bioactivities, has received lesser attention [14]. Aside from the mutagenicity and genotoxicity studies by Chen et al. [8], bioactivities of mycelium and culture broth of *L. rhinocerotis* have not been evaluated. In this study, we focused on the comparative analyses of bioactivities and chemical profiling of *L. rhinocerotis* from different morphological/developmental stages (mycelium and sclerotium) and culture conditions (shaken and static cultures) of liquid fermentation. The potential of the mycelium and culture broth as substitutes for the sclerotium is discussed.

## Materials and Methods

### Mushroom cultivation

The axenic culture of *L. rhinocerotis* (KUM61075) was obtained from the Mushroom Research Centre, University of Malaya. The sclerotium of *L. rhinocerotis* was produced by solid-substrate fermentation of mycelium on agroresidues according to the method of Abdullah et al. [7]. Harvested sclerotium was washed with distilled water and dried in the oven at 40°C for 3−5 days. The glucose-yeast extract-malt extract-peptone (GYMP, Oxoid, Hampshire, UK) medium was used for liquid fermentation [2]. Flasks were inoculated with mycelial plugs and incubated at 25°C under static conditions or placed on a reciprocal shaker at 150 rpm. After 15 days, the cultures were harvested; mycelium was filtered off from the culture broth and repeatedly washed with distilled water. Mycelium and culture broth were freeze-dried and kept in air-tight containers at −20°C.

### Preparation of aqueous methanol extracts

**Figure 1:**
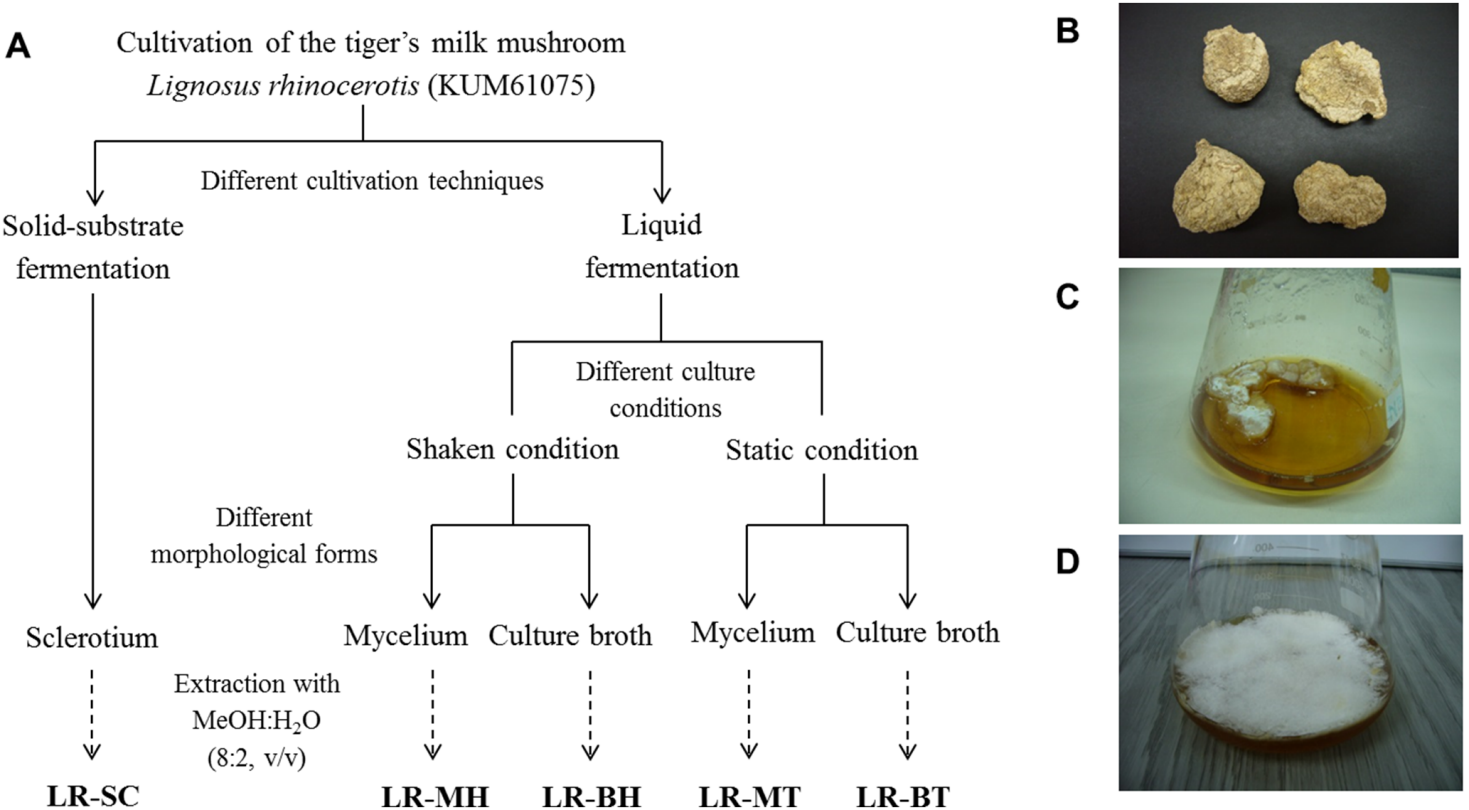
Overview of experimental design. (A) Cultivation of *Lignosus rhinocerotis* and extraction of low-molecular-weight compounds using aqueous methanol. Extracts were prepared from the mycelium (LR-MH, shaken cultures; LR-MT, static cultures), culture broth (LR-BH, shaken cultures; LR-BT, static cultures), and sclerotium (LR-SC). The different developmental/morphological forms of *L. rhinocerotis*: (B) sclerotium from solid-substrate fermentation, (C) mycelial pellet in shaken cultures, and (D) mycelial pellicle in static cultures of liquid fermentation.

Mushroom samples were ground to a fine powder using a Waring blender. The powdered mycelium and sclerotium as well as the freeze-dried culture broth were soaked in 80% (v/v) methanol (analytical grade) in water at a ratio of 1∶20 (w/v) for 3 days. The extract was then decanted and filtered through Whatman No. 1 filter paper, and the residues were re-extracted twice. The filtrates were combined, and excess solvent was removed under pressure at 40°C using a rotary evaporator, producing five brownish extracts: LR-MH (mycelium from shaken conditions); LR-MT (mycelium from static conditions); LR-BH (culture broth from shaken conditions); LR-BT (culture broth from static conditions); and LR-SC (sclerotium). The extracts were kept at −20°C prior to analyses. A summary of the different cultivation techniques, culture conditions of liquid fermentation, and extraction procedures involved is depicted in Figure 1.

### Evaluation of antioxidant capacity of the extracts

The antioxidant capacity of *L. rhinocerotis* extracts was evaluated based on methods previously reported (below); hence, only the necessary modifications will be indicated. Standards including quercetin dihydrate, 6-hydroxy-2,5,7,8-tetramethylchromane-2-carboxylic acid (Trolox), ferrous sulphate heptahydrate (FeSO_4_·7H_2_O), and disodium ethylenediamine tetraacetate (Na_2_EDTA) were obtained from Sigma-Aldrich (St. Louis, USA), while 1,1,3,3-tetraetoxypropane (TEP) (the tetraethylacetal of malondialdehyde [MDA]) was purchased from Merck (Darmstadt, Germany). Other chemicals and solvents used were of analytical grade. All extracts were dissolved in 50% (v/v) methanol in water to produce stock solutions of 20 mg/ml and diluted to desired concentrations for the following assays:

#### 2,2-Diphenyl-1-picrylhydrazyl (DPPH) free-radical-scavenging activity

The ability of the extracts to scavenge DPPH free radicals was measured according to methods of Kong et al. [15]. The results were expressed in terms of IC_50_ values (the concentration of extract required to produce 50% inhibition).

#### 2,2′-Azino-bis(3-ethylbenzthiazoline-6-sulphonic acid) (ABTS) radical-scavenging activity

The ABTS radical-scavenging activity of the extracts was evaluated based on the method of Re et al. [16]. The results were expressed as mmol Trolox equivalent/g extract.

#### Ferric-reducing antioxidant power (FRAP) assay

The FRAP assay was performed according to Benzie and Strain [17] with modifications, in which 10 µl of extracts were mixed with 300 µl of freshly prepared FRAP reagent. The results were expressed as µmol FeSO_4_·7H_2_O equivalent/g extract.

#### Cupric ion-reducing antioxidant capacity (CUPRAC) assay

The CUPRAC assay was performed based on the method by Ribeiro et al. [18]. The results were expressed as µmol Trolox equivalent/g extract.

#### Metal-chelating activity

The ability of the extracts to chelate metal ions was analysed using the method by Jimenez-Alvarez et al. [19] with modifications. Briefly, 50 µl of extracts and 50 µl of 100 µM FeCl_2_ were mixed. After 20 min of incubation, 50 µl of 100 µM ferrozine were added to the mixture. The results were expressed as µmol Na_2_EDTA equivalent/g extract.

#### Inhibition of lipid peroxidation

The inhibitory effect of the extracts against lipid peroxidation was determined based on a method to measure thiobarbituric-acid-reactive substances (TBARS) in FeSO_4_-induced lipid peroxidation in egg yolk homogenates [20] with minor modifications. The concentration of FeSO_4_ used was 20 mM. The results were expressed as TEP equivalent/g extract.

### Cell culture

The following cell lines were purchased from the American Type Culture Collection (ATCC, Manassas, VA, USA): A549 (human lung carcinoma); Caco-2; HCT 116; HT-29 (human colorectal carcinoma); Chang Liver (HeLa derivative); HEK-293 (human embryonic kidney); Hep G2 (human hepatocellular carcinoma); HL-60 (human acute promyelocytic leukemia); MCF7, MDA-MB-231 (human breast adenocarcinoma); MCF 10A (human breast epithelial); NRK-52E (rat kidney epithelial); PC-3 (human prostate adenocarcinoma); RAW 264.7 (mouse leukemic monocyte macrophage); Vero (African green monkey kidney epithelial); WRL 68 (HeLa derivative); and 4T1 (mouse mammary gland carcinoma). The HSC-2 (human oral squamous carcinoma) line was obtained from the Human Science Research Resources Bank (Japan), and HK1 (human nasopharyngeal carcinoma) was a gift from Professor Tsao at the University of Hong Kong. The OKF6 (immortalised human oral epithelial) and NP 69 (immortalised human nasopharyngeal epithelial) lines were obtained from the BWH Cell Culture and Microscopy Core at the Harvard Institutes of Medicine (USA) and University of Hong Kong Culture Collections, respectively.

Cell culture media and supplements were purchased from Gibco Invitrogen (Life Technologies, USA) unless otherwise stated. The A549, HT-29, HCT 116, HL-60, MCF7, PC-3, and 4T1 lines were maintained in RPMI-1640 media; Chang Liver, HEK-293, Hep G2, MDA-MB-231, NRK-52E, RAW 264.7, Vero, and WRL 68 were maintained in DMEM; while Caco-2, HK1, and HSC-2 were grown in MEM. All media were supplemented with 10% (v/v) heat-inactivated fetal bovine serum (FBS) and 100 units/ml of penicillin/streptomycin. The NP 69 and OKF6 lines were cultured in keratinocyte serum-free media (Keratinocyte-SFM, Invitrogen) supplemented with L-glutamine, human epidermal growth factor (hEGF, 0.1 ng/ml), bovine pituitary extract (BPE, 50 µg/ml), and Ca^2+^ (final concentration, 0.3 mM). The MCF 10A line was grown in serum-free mammary epithelial growth media (MEGM BulletKit, Lonza, USA). The basal medium was supplemented with BPE (50 µg/ml), hydrocortisone (0.5 µg/ml), hEGF (10 ng/ml), insulin (5 µg/ml), and cholera toxin (100 ng/ml). Cells were cultured in a 5% CO_2_ incubator at 37°C in a humidified atmosphere.

### Evaluation of cytotoxic effect of the extracts

The effect of the extracts on cell viability was evaluated using the 3-(4,5-dimethylthiazol-2-yl)-2,5-diphenyltetrazolium bromide (MTT) (Sigma-Aldrich, USA) assay, as previously described [21]. All extracts were dissolved in 50% (v/v) dimethyl sulphoxide (DMSO) in water to produce stock solutions of 50 mg/ml, which were further diluted with culture media to desired concentrations. Cells (3−5×10^3^ cells/well) were seeded and allowed to attach overnight prior to treatment with extracts at final concentrations of 20 and 200 µg/ml. The percentage of cell viability after 72 h of incubation was determined by the following equation:
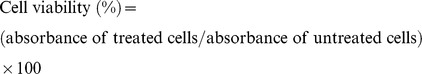



### Determination of chemical composition of the extracts

Total sugars were determined using the phenol-sulphuric assay [22]. D-glucose (Merck) was used as the standard. Protein content was analysed using the Pierce Coomassie Plus (Bradford) Protein Assay (ThermoScientific, Massachusetts, USA) according to the manufacturer’s protocol, with bovine serum albumin as the standard. The level of phenolics was estimated using the Folin-Ciocalteu reagent [23] with gallic acid (Sigma-Aldrich, USA) as the standard.

### Electrophoretic analysis of proteins

Sodium dodecyl sulphate-polyacrylamide gel electrophoresis (SDS-PAGE) of the extracts was carried out using 16% (w/v) separating and 5% (w/v) stacking gels in a vertical slab gel apparatus (C.B.S. Scientific Company, Inc., California, USA), as previously reported [21]. Bands were visualised by Coomassie Brilliant Blue R-250 (Sigma-Aldrich) and silver staining.

### Chromatographic and mass-spectrometric analyses

#### SELDI-TOF-MS

For the surface-enhanced-laser-desorption-ionisation time-of-flight mass spectrometry (SELDI-TOF-MS) analysis, extracts were spotted on the reverse-phase or hydrophobic H50 ProteinChip arrays and analysed using a ProteinChip SELDI System PSC 4000 (Bio-Rad Laboratories, Inc., California, USA), as previously described [9], [21].

#### GC-MS

The gas chromatography-mass spectrometry (GC-MS) analysis was performed using a 6890 N gas chromatograph (Agilent Technologies, Inc., California, USA) equipped with a 5975 Mass Selective Detector. The HP-5 MS (5% phenylmethylsiloxane) capillary column (30.0 m×25 mm×25 µm) was initially set at 70°C, increased to 300°C, and then held for 10 min. Helium was used as the carrier gas at flow rate of 1 ml/min. The total ion chromatogram (TIC) was auto-integrated by ChemStation. Chemical constituents were identified by comparison with the accompanying spectral database (NIST 2011, Mass Spectral Library, USA) and literature, where applicable.

#### UHPLC-ESI-MS/MS

The analysis was performed using a Flexar FX15 ultra high-performance liquid chromatograph (UHPLC, PerkinElmer, Inc., Massachusetts, USA) coupled with an AB SCIEX 3200 QTrap hybrid linear ion trap triple-quadruple mass spectrometer equipped with a turbo ion spray source. Chromatographic separation was achieved on a Phenomenex Aqua C18 (5 µm, 50 mm×2 mm) column. Mobile phase A was composed of water with 0.1% (v/v) formic acid and 5 mM ammonium formate, whereas the mobile phase B consisted of acetonitrile containing 0.1% (v/v) formic acid and 5 mM ammonium formate. Elution was performed by means of a linear gradient from 10−90% B (0−8 min) held for 3 min, returned to 10% B in 0.1 min, and then re-equilibrated for 4 min before the next injection. Ionisation was achieved via electrospray ionisation on the AB Sciex Turbo V source with an ionisation temperature of 500°C and purified nitrogen gas (99%) as the collision gas via nebulisation. Collision energy was set at 35 eV for mass-fragmentation purposes. Full scan with MS/MS data collection analyses was performed in negative mode. Data analysis, processing, and interpretation were carried out using the AB SCIEX Analyst 1.5 and Advanced Chemistry Development, Inc., (ACD/Labs, Ontario, Canada) MS Processor software. MarkerView Software (AB SCIEX, Massachusetts, USA) was used for principal component analysis (PCA). The following parameters were used for PCA: retention time (R*_T_*) range: 0−15 min, R*_T_* tolerance: 0.5 min, mass range: m/z 100−1000, mass tolerance: 0.01 Da, and noise threshold: 5.

### Statistical analysis

Analysis was performed in triplicates. Results were expressed as means ± standard deviation (SD) or standard error (SE). The data were statistically analysed using the IBM SPSS Statistics Version 19 software (SPSS Inc., New York, USA). All mean values were analysed by one-way analysis of variance (ANOVA) followed by Tukey’s Honestly Significant Difference (*p*<0.05) to detect significance between groups.

## Results and Discussion

### Yields of mushroom samples

Liquid fermentation is routinely used for production of mushroom mycelia and metabolites. The yields (g/100 ml) of the freeze-dried culture broth (LR-BH: 2.94, LR-BT: 3.30) were higher than that of mycelium (LR-MH: 0.62, LR-MT: 0.59) regardless of culture conditions of liquid fermentation. Although noted as a slow-growing species [7], the yield of the mycelium of *L. rhinocerotis* was higher than the maximum yield of the mycelium of an edible mushroom (*Agaricus brasiliensis*) cultured by shaken flasks (1.02 g/100 ml) [24] and an medicinal mushroom (*Ganoderma lucidum*) cultured in static flasks (1.25 g/100 ml) [14]; however, the culture conditions, such as media composition and aeration, were different, and these could have affected fungal growth. On the other hand, the yield of sclerotium (on a dry-weight basis) was 1.3−2.0 g/g substrate [7].

### Yields of mushroom extracts

A mixture of methanol and water was used to widen the spectrum of extracted low-molecular-weight constituents, especially compounds with higher polarity, such as phenolic compounds [24]. The yields (w/w) of the aqueous methanol extracts (in descending order) were LR-BT (75.1%) > LR-BH (69.9%) > LR-MT (21.0%) > LR-MH (14.7%) > LR-SC (2.7%). The higher yields of LR-BH and LR-BT, compared to other extracts, indicated that constituents in the culture broths were readily soluble in aqueous methanol. Our results were consistent with previous findings where the yield of culture broth was higher than that of mycelia and/or fruiting bodies [25], [26]. The yield of LR-SC was the lowest, as the sclerotium of *L. rhinocerotis* was reported to predominantly consist of carbohydrates [1], [2], such as dietary fibres, that are insoluble in the extraction solvent used in this study.

### Comparative antioxidant capacity

Antioxidants confer protection against cellular damage caused by oxidative stress and thus potentially ameliorate diseases, such as cancer, diabetes, and cardiovascular and neurodegenerative disorders. The medicinal properties of *L. rhinocerotis* might be partially associated with its antioxidant capacity. In this study, several assays based on different antioxidant mechanisms were employed to assess the antioxidant capacity of the extracts. The free radical-scavenging activities, reducing properties, metal-chelating activities, and inhibitory effects on lipid peroxidation by the extracts of *L. rhinocerotis* are shown in Table 1. Overall, the antioxidant capacity of the mycelium and culture broth of *L. rhinocerotis* was found to be either higher or comparable to that of the sclerotium; however, the relative potency of the five extracts, in different assays, was not consistent. For radical scavenging, the extracts exhibited varying degrees of DPPH free-radical-scavenging activities with extracts of the mycelium, and sclerotium (IC_50_: 0.9−3.6 mg/ml) showed stronger scavenging activities than those of the culture broth (IC_50_: 4.2−6.9 mg/ml). The ability of the extracts to quench the ABTS radicals was comparable, but the activity decreased in the order of culture broth > sclerotium > mycelium. The reducing properties of the extracts were measured using the FRAP and CUPRAC assays. In the FRAP assay, LR-BH, LR-BT, and LR-MH showed higher reducing properties (67.0−85.7 µmol FeSO_4_·7H_2_O equivalent/g extract) than other extracts. The reducing properties of the extracts as measured by the CUPRAC assay revealed a trend consistent with the FRAP assay, in that LR-MH, LR-BH, and LR-BT also exhibited higher activities (268.0−350.4 µmol Trolox equivalent/g extract). Through the Fenton reactions, hydroxyl radicals generated by transition metals could stimulate lipid peroxidation. By stabilising transition metals, chelating agents might impair the production of free radicals. The metal-chelating activity of the extracts ranged from 26.8−59.4 µmol Na_2_EDTA equivalent/g extract. The LR-BT exhibited the highest ferrous-chelating activity, comparable to LR-SC. On the other hand, the level of MDA was taken as an indicator of lipid peroxidation, where a lower concentration of MDA reflects a higher inhibitory potential. The inhibitory potentials of the extracts against FeSO_4_-induced lipid peroxidation were comparable to each other except for LR-BT, in which the MDA level was significantly lower (*p*<0.05).

**Table 1:** Antioxidant capacity of *L. rhinocerotis* extracts.

Extracts	*DPPH(IC_50_, mg/ml)	TEAC(mmol troloxequiv./g extract)	FRAP(µmol FeSO_4_·7H_2_Oequiv./g extract)	CUPRAC(µmol Trolox equiv./gextract)	Metal chelating(µmol Na_2_EDTAequiv./gextract)	Inhibition of lipidperoxidation(mmol MDA/g extract)
LR-MH	0.94±0.01 a	143±13.42 a	71.25±1.91 a	350.41±5.15 a	40.44±0.07 a	1.51±0.08 a
LR-MT	3.72±0.11 b	128.43±9.25 a	21.21±1.04 b	214.33±11.66 b, c	31.97±1.68 b	1.55±0.05 a
LR-BH	4.23±0.08 c	186.67±7.54 a, b	67.02±3.00 a	274.78±7.34 d	26.76±0.50 c	1.89±0.09 a
LR-BT	6.87±0.06 d	223.05±8.26 b	85.73±4.02 c	268.01±5.61 a, d	59.43±0.36 d	1.48±0.04 b
LR-SC	3.60±0.10 b	162.93±24.63 a, b	23.01±1.31 b	192.53±15.86 b	57.95±0.14 d	1.59±0.02 a

The extracts were dissolved in 50% (v/v) methanol in water for the antioxidant assays. Results were expressed as mean ± SE of at least three independent experiments (*n* = 3–5) performed in triplicates. The different letters (a–d) within a column represent means with significance difference (*p*<0.05).

*Quercetin dihydrate (IC_50_: 0.091 mg/ml) was used as the positive control in the DPPH free radical scavenging assay.

Previous investigations found that no firm conclusions regarding the relative antioxidant capacity of mushroom samples from different morphological/developmental stages and cultivation techniques, such as the fruiting body, mycelium, culture broth, and/or sclerotium. A direct comparison of values obtained from antioxidant capacity evaluation assays performed in different laboratories is not possible due to the differences in methodologies used. In addition, comparative analyses on the antioxidant capacity of mushrooms from different morphological/developmental stages are scarce, and, in most cases, findings are inconsistent. For instance, according to Reis et al. [27], the fruiting bodies of several cultivated mushrooms generally revealed higher antioxidant properties than the corresponding mycelia. In a separate report on *A. brasiliensis*, Carvajal et al. [24] found that mycelial extracts exhibited stronger ABTS radical-scavenging and ferrous ion-chelating abilities but weaker DPPH free-radical scavenging and inhibition of lipid peroxidation than the fruiting body. Wong et al. [28] observed that the mycelial extract (consisting of both mycelium and culture broth) of *Hericium erinaceus* showed stronger reducing capacity than the fruiting bodies as determined by the FRAP assay, but the extract’s ability to scavenge DPPH free radicals was lower. When comparisons are made between fruiting bodies and mycelia, other factors such as mushroom strain, cultivation techniques, culture conditions, and postharvest processing should be considered. As indicated, most studies focused on the comparison between fruiting bodies and mycelia, and sclerotia received lesser attention. One plausible explanation for this is that very few sclerotia-producing mushrooms are commercially available. Since mushroom sclerotia are, in general, predominantly carbohydrates, the antioxidant capacity of different types of sclerotial polysaccharides have been extensively studied, e.g. the water- and alkaline-soluble polysaccharides from *Pleurotus tuber-regium* [29] and *Inonotus obliquus* [30]; however, antioxidants in the form of low-molecular-weight constituents remain poorly investigated.

### Comparative cytotoxic effect

Earlier, Chen et al. [8] reported that the mycelium of *L. rhinocerotis* did not provoke mutagenicity and genotoxicity; however, its cytotoxicity in mammalian cells was not evaluated. In light of this, extracts were screened for cytotoxicity against a panel of 21 mammalian cell lines using the MTT assay. According to the U.S. National Cancer Institute, crude extracts with IC_50_ values less than 20 µg/ml, after an incubation period of 48−72 h, are considered active [31]. As shown in Table 2, cellular viability of most cells was maintained above 70% following treatment with 20 µg/ml of extracts; hence, the extracts were considered non-cytotoxic. At higher concentration (200 µg/ml), some of the non-tumourigenic cells, usually used as models of normal cells in cytotoxicity evaluation, were found to be more susceptible than the corresponding solid tumours. For instance, NP 69 and OKF6 were observed to be more susceptible to the extracts than HK1 and HSC-2, respectively; hence, this implied non-selective cytotoxicity of the extracts against these cell lines.

**Table 2:** Cytotoxic effect of *L. rhinocerotis* extracts.

Cell lines and extracts	Cell viability (%) after 72 h of incubation (Mean ± SE)									
	LR-MH		LR-MT		LR-BH		LR-BT		LR-SC	
Concentration (µg/ml)	20	200	20	200	20	200	20	200	20	200
A549	98.1±2.37	94.3±5.03	82.5±2.46	67.0±2.70	101.0±1.10	99.5±2.28	105.2±4.85	89.2±2.94	98.5±5.33	80.0±1.73
Caco-2	99.2±2.42	91.6±3.02	97.1±2.07	79.3±2.89	95.0±0.94	79.9±3.25	98.4±5.46	84.1±1.16	98.6±3.17	76.9±1.56
Chang Liver	90.4±0.89	99.0±1.78	101.1±1.23	93.0±1.13	103.4±0.77	99.5±0.97	102.5±2.07	98.0±1.35	100.6±3.89	88.8±0.71
HCT 116	110.0±4.09	92.2±1.68	93.7±0.64	81.0±3.38	111.2±5.9	107.7±1.24	111.6±0.33	104.3±0.14	92.2±4.10	90.5±1.74
HEK-293	99.9±3.32	85.5±3.25	102.3±1.69	76.6±1.21	93.8±3.79	70.7±2.20	94.2±6.77	69.8±5.49	94.9±6.23	48.1±0.17
Hep G2	104.1±0.63	100.1±1.16	99.8±0.44	88.6±2.22	102.1±2.53	90.0±2.54	100.1±2.05	103.9±1.17	88.5±3.79	70.6±2.23
HK1	91.2±5.70	89.4±5.08	92.0±1.10	93.0±1.88	92.8±4.40	86.5±9.41	94.6±1.58	93.1±5.29	95.4±0.44	88.3±7.21
HL-60	101.0±1.63	83.3±0.84	108.1±1.97	96.4±1.87	119.6±3.86	100.9±2.11	124.2±3.03	101.1±1.31	106.2±0.43	91.0±2.43
HT-29	111.4±2.75	110.8±7.96	106.2±1.64	71.6±3.87	114.1±2.17	108.7±3.09	124.0±5.33	113.1±1.61	115.5±2.63	105.3±4.94
HSC2	98.2±3.71	90.4±0.94	101.3±2.09	94.5±1.53	90.9±0.88	91.1±1.27	95.0±1.25	97.7±2.11	92.6±1.34	95.8±1.87
MCF7	91.6±5.72	92.1±1.01	92.7±4.88	85.7±3.03	83.3±2.95	77.5±2.51	104.7±0.08	95.6±3.50	114.6±3.39	108.8±3.12
MCF 10A	110.3±3.64	81.4±1.95	102.1±2.57	37.1±4.34	100.6±6.95	107.3±3.23	102.3±6.54	83.2±4.69	93.8±3.95	94.0±2.22
MDA-MB-231	109.1±0.19	89.8±1.80	109.7±3.44	77.5±0.17	102.6±0.71	85.0±3.24	102.2±2.40	82.1±1.41	97.5±3.57	73.0±4.33
NP 69	81.2±0.87	65.4±0.20	79.6±0.74	62.1±2.29	79.6±0.68	67.5±1.97	79.1±0.82	77.5±0.69	60.3±0.42	56.01±0.38
NRK-52E	107.9±5.02	84.2±2.82	102.4±1.29	75.5±2.21	113.9±1.60	97.6±1.23	112.9±3.40	113.9±1.33	109.2±3.95	102.2±0.71
OKF6	93.7±0.76	68.3±0.16	89.0±1.11	65.6±2.83	105.6±2.19	103.1±1.69	105.7±0.48	105.4±0.57	78.0±1.86	70.7±0.67
PC3	91.2±3.29	89.4±2.93	92.0±0.64	93.0±1.09	92.8±2.54	86.5±5.43	94.6±0.91	93.1±3.05	95.4±0.25	88.3±4.16
RAW 264.7	99.9±4.66	88.8±3.82	97.4±2.00	75.4±0.58	103.9±1.03	107.8±4.26	102.3±3.73	94.7±3.22	93.1±3.64	64.1±2.19
Vero	101.3±1.85	94.0±2.24	85.8±1.00	84.6±1.66	118.7±1.43	117.2±2.55	123.3±4.21	132.7±0.64	90.6±2.57	80.6±3.15
WRL 68	104.3±3.68	105.8±3.32	91.8±2.89	83.7±0.68	98.5±0.11	102.7±0.42	97.8±0.19	104.7±1.21	94.7±1.79	90.4±1.81
4T1	98.9±4.35	92.7±4.07	89.7±0.76	69.9±1.16	84.2±3.35	68.8±2.23	87.6±5.44	86.9±6.07	72.6±2.87	61.1±1.52

The extracts were dissolved in 50% (v/v) dimethyl sulphoxide (DMSO) in water and diluted in media for the MTT assay. The final concentration of DMSO in the well was less than 0.5% (v/v) and this did not affect cell viability. Results were expressed as mean ± SE of three independent experiments (*n* = 3) performed in triplicates.

Our results also indicated that the extracts of the mycelium and culture broth of *L. rhinocerotis* showed mild cytotoxic effects against most cell lines, comparable to the sclerotium extract. LR-MT (200 µg/ml) was noted to exert relatively strong cytotoxicity against MCF 10A (cell viability: 37.1%) compared to MCF7 (85.7%) and MDA-MB-231 (77.5%); however, other extracts (LR-MH, LR-BH, LR-BT, LR-SC) did not affect the viability of MCF 10A (>80%), and this is likely to indicate presence of cytotoxic metabolites that might be found only in LR-MT. This is the first attempt to screen for cytotoxicity in the extracts of *L. rhinocerotis* from liquid fermentation (i.e., mycelium and culture broth). Earlier, the cytotoxic effects of alcoholic extracts of *L. rhinocerotis* sclerotium were studied, albeit there was slight variation in the methodology used (e.g., solvent and extraction techniques, cell lines, and duration of treatment). In a previous study by Eik et al. [11], an ethanol extract of the sclerotium of the *L. rhinocerus* TM02 cultivar was reported to exert low toxicity (IC_50_: 282.1 µg/ml) against PC-12 cells (rat pheochromocytoma) after 48 h treatment. Similarly, an aqueous methanol extract of a wild-type *L. rhinocerotis*, prepared using a pressurised liquid extraction method, showed weak cytotoxicity (IC_50_: 600 µg/ml) against HCT 116 and no effect (IC_50_>2000 µg/ml) against CCD-18Co (human colon fibroblast) cells after 24 h treatment [13]. Therefore, the alcoholic extracts of the sclerotium of *L. rhinocerotis* (including LR-SC from this study), in general, were non-cytotoxic (IC_50_>20 µg/ml) against mammalian cells. On the other hand, the cold aqueous extracts showed relatively high cytotoxicity based on previous findings. Cytotoxic components in the sclerotium of *L. rhinocerotis* were suspected to be mainly heat-labile protein/peptide(s) [21] and/or high-molecular-weight, protein-carbohydrate complexes [12], rather than the low-molecular-weight constituents.

### Chemical composition

Table 3 shows the chemical characterisation of the extracts of *L. rhinocerotis*. The extracts contained relatively low concentrations of sugars and proteins. In aqueous methanol, the solubility of polysaccharides and proteins is low, but simple compounds (e.g., sugars, amino acids, and peptides) can be dissolved [24]. Although the level of sugars in LR-SC was the lowest, its protein content was significantly higher than others. The concentration of phenolics in the extracts ranged from 7.9−18.8 mg gallic acid equivalent/g extract. Interestingly, the mycelium and culture broth from shaken cultures contained significantly higher phenolics than their counterparts from static cultures.

**Table 3:** Chemical composition of *L. rhinocerotis* extracts.

Extracts	Total sugars(mg glucose/g extract)	Total proteins(mg protein/g extract)	Total phenolic content(mg GAE/g extract)
LR-MH	185.8±3.91 a	3.4±0.08 a	18.8±1.49 a
LR-MT	413.9±41.6 b	4.5±0.15 b	7.9±2.12 b
LR-BH	267.9±22.6 c	3.7±0.13 c	15.3±1.12 a, c
LR-BT	267.9±22.6 d	1.9±0.11 d	11.8±1.14 b, c
LR-SC	118.1±16.51 a, d	7.4±0.07 e	13.2±2.41 c

Results were expressed as mean ± SD of triplicate measurements. The different letters (a–d) within a column represent means with significance difference (*p*<0.05).

### Protein profiling

**Figure 2:**
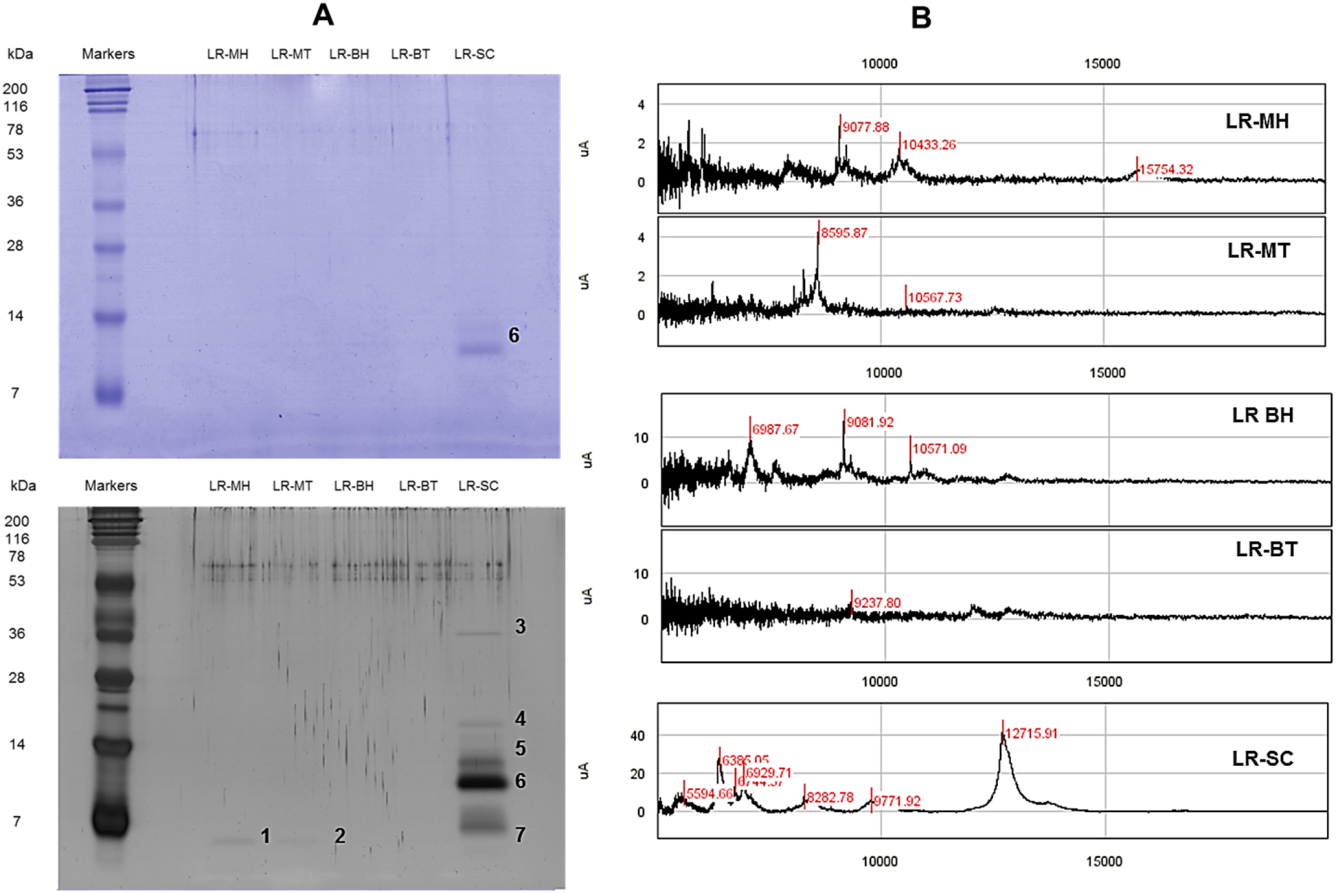
Protein profiling. (A) Electrophoretic analysis of proteins in the extracts of *Lignosus rhinocerotis* and visualisation by Coomassie Brilliant Blue (top) and silver staining (bottom). Molecular weight (MW) of the bands was estimated from the plot of log MW vs. relative migration distance (R_f_) based on the values obtained from the bands of the marker (7−200 kDa). The estimated sizes of the bands were as follows: 1, 2 (4.0 kDa), 3 (38.0 kDa), 4 (14.0 kDa), 5 (9.5 kDa), 6 (8.0 kDa), and 7 (4.7 kDa). (B) Representative SELDI-TOF-MS spectra of the low-molecular-weight proteins (5−20 kDa) in the extracts. The x-axis represents the m/z values, and the y-axis represents the intensity of the signals (µA). Peaks with signal/noise ratios (S/N) >5 were automatically detected.

The higher protein content in LR-SC compared to other extracts was confirmed with further protein profiling (Figure 2). Results from SDS-PAGE showed that LR-SC was characterised by a single band (approximately 8 kDa) that could be visualised after Coomassie blue staining. Silver staining, a more sensitive visualisation technique, revealed the presence of other proteins, presumably those of lower abundance. These include a faint band (approximately 5 kDa) common to LR-MH and LR-MT and additional bands (5−40 kDa) in LR-SC. Compared to our previous work [21], LR-SC lacked most of the proteins present in the cold aqueous extract of *L. rhinocerotis*. The SELDI-TOF-MS analysis was performed to detect low-molecular-weight proteins that might have been resolved poorly on the gel. The number of peaks in the extracts and their intensities were low. Most peaks were in the range of 15 kDa or less. The SELDI-TOF-MS spectrum of LR-SC was different from that of LR-MH, LR-MT, LR-BH, and LR-BT; however, the profile showed some resemblance to that of the cold aqueous extract of *L. rhinocerotis*, as previously reported [21].

### Identification of chemical constituents by GC-MS

By using GC-MS, several low-molecular-weight compounds composed of sugars and their derivatives, fatty acids and their methyl esters, cyclic peptides, sterols, and amides in the extracts of *L. rhinocerotis* were identified (Tables 4−6, Figures S1−S5). The LR-MH and LR-MT were characterised by the presence of 9,12-octadecadienoic acid (Z,Z) (linoleic acid) (11.7−14.5%), its methyl ester (2.6−11.2%), and a derivative of its ethyl ester, 9,12-octadecadienoic acid (Z,Z)-,2-hydroxy-1-(hydroxymethyl)ethyl ester (2-monolinolein) (13.3−17.0%). Both extracts also contained n-hexadecanoic acid (palmitic acid) (6.1−7.1%) and its methyl ester (1.2−6.5%); however, hexadecanamide (palmitic amide) was detected only in LR-MT. Some compounds were found only in LR-MT, such as arabinitol, octadecanoic acid (stearic acid), and ergosterol.

**Table 4:** Chemical constituents in LR-MH and LR-MT based on GC-MS analysis.

R_T_ (min)	Compounds	Molecular formula	Molecular weight	Area (%)	
				LR-MH	LR-MT
12.12	Methyl β-D-galactopyranoside	C_7_H_14_O_6_	194.18	8.16	ND
12.97	Arabinitol	C_5_H_12_O_5_	152.15	ND	4.46
13.51	Pyrrolo[1,2-a]pyrazine-1,4-dione, hexahydro-	C_7_H_10_N_2_O_2_	154.17	8.36	10.78
14.91	Hexadecanoic acid, methyl ester	C_17_H_34_O_2_	270.45	1.23	6.50
15.30	n-Hexadecanoic acid	C_16_H_32_O_2_	256.42	6.14	7.10
16.56	9,12-Octadecadienoic acid (Z,Z)-, methyl ester	C_19_H_34_O_2_	294.47	2.63	11.23
16.97	9,12-Octadecadienoic acid (Z,Z)-	C_18_H_32_O_2_	280.45	11.73	14.52
17.19	Octadecanoic acid	C_18_H_36_O_2_	284.48	ND	1.25
17.39	Hexadecanamide	C_16_H_33_NO	255.44	ND	1.40
18.35	11,13-Eicosadienoic acid, methyl ester	C_21_H_38_O_2_	322.53	ND	0.53
18.70	Cyclopentadecanone, 2-hydroxy-	C_15_H_28_O_2_	240.38	ND	0.23
18.93	9-Octadecenamide, (Z)-	C_18_H_35_NO	281.48	4.76	3.54
21.51	9,12-Octadecadienoic acid (Z, Z)-, 2-hydroxy-1-(hydroxymethyl)ethyl ester	C_21_H_38_O_4_	354.52	13.26	16.98
22.60	2,3-Dihydroxypropyl elaidate	C_21_H_40_O_4_	356.54	0.28	0.27
25.69	Ergosterol	C_28_H_44_O	396.65	ND	1.30

Area (%) was determined based on the TIC of LR-MH (Figure S1) and LR-MT (Figure S2). Identification of the compounds was based on mass spectral analysis. R*_T_*, retention time; ND, not detected.

**Table 5:** Chemical constituents in LR-BH and LR-BT based on GC-MS analysis.

R*_T_* (min)	Compounds	Molecular formula	Molecular weight	Area (%)	
				LR-BH	LR-BT
3.61	2-Furancarboxaldehyde, 5-methyl-	C_6_H_6_O_2_	110.11	0.89	0.65
4.46	Benzeneacetaldehyde	C_8_H_8_O	120.15	1.24	0.51
6.70	1,4∶3,6-Dianhydro-α-D-glucopyranose	C_6_H_8_O_4_	144.13	2.08	ND
11.16	β-D-glucopyranose, 1,6-anhydro	C_6_H_10_O_5_	162.14	0.80	ND
13.68	Pyrrolo[1,2-a]pyrazine-1,4-dione, hexahydro-	C_7_H_10_N_2_O_2_	154.17	15.60	20.12
15.19	Pyrrolo[1,2-a]pyrazine-1,4-dione, hexahydro-3-(2-methylpropyl)-	C_11_H_18_N_2_O_2_	210.27	ND	4.18
15.23	n-Hexadecanoic acid	C_16_H_32_O_2_	256.42	2.86	ND
16.95	9,12-Octadecadienoic acid (Z,Z)-	C_18_H_32_O_2_	280.45	2.63	ND
17.36	Hexadecanamide	C_16_H_33_NO	255.44	2.50	ND
18.93	9-Octadecenamide, (Z)-	C_18_H_35_NO	281.48	7.43	3.19
19.25	Pyrrolo[1,2-a]pyrazine-1,4-dione, hexahydro-3-(phenylmethyl)-	C_14_H_16_N_2_O_2_	244.29	ND	1.07

Area (%) was determined based on the TIC of LR-BH (Figure S3) and LR-BT (Figure S4). Identification of the compounds was based on mass spectral analysis. R*_T_*, retention time; ND, not detected.

**Table 6:** Chemical constituents in LR-SC based on GC-MS analysis.

R*_T_* (min)	Compounds	Molecular formula	Molecular weight	Area (%) LR-SC
12.87	D-glucopyranoside, methyl	C_7_H_14_O_6_	194.18	32.17
13.55	Pyrrolo[1,2-a]pyrazine-1,4-dione, hexahydro	C_7_H_10_N_2_O_2_	154.17	3.51
15.28	n-Hexadecanoic acid	C_16_H_32_O_2_	256.42	3.31
16.43	Oleic acid	C_18_H_34_O_2_	282.46	0.24
16.94	9,12-Octadecadienoic acid (Z,Z)-	C_18_H_32_O_2_	280.45	4.71
17.15	Octadecanoic acid	C_18_H_36_O_2_	284.49	1.04
18.91	9-Octadecenamide, (Z)-	C_18_H_35_NO	281.48	1.56
25.68	Ergosta-4,7,22-trien-3β-ol	C_28_H_44_O	396.65	5.31

Area (%) was determined based on the TIC of LR-SC (Figure S5). Identification of the compounds was based on mass spectral analysis. R*_T_*, retention time; ND, not detected.

On the other hand, pyrrolo[1,2-a]pyrazine-1,4-dione, hexahydro or cyclo(leucyloprolyl) (15.6−20.1%) and 9-octadecanamide (7.4−3.2%) were the major compounds in LR-BH and LR-BT. Another two cyclic peptides were present only in LR-BT. These were pyrrolo[1,2-a]pyrazine-1,4-dione, hexahydro-3-(2-methylpropyl)- or cyclo(D-phenylalanyl-L-prolyl) (4.2%) and pyrrolo[1,2-a]pyrazine-1,4-dione,hexahydro-3-(phenylmethyl)- or cyclo(phenylalanylprolyl) (1.1%). Several compounds identified from the mycelial extracts (e.g., n-hexadecanoic acid and hexadecanamide) were also found in LR-BH. In addition, sugars and their derivatives, such as 1,4∶3,6-dianhydro-α-D-glucopyranose and β-D-glucopyranose, 1,6-anhydro (levoglucosan) were detected.

The LR-SC was characterised having methyl D-glucopyranoside, a glucoside, as the major component (45.3%) as well as ergosta-4,7,22-trien-3β-ol (5.3%), linoleic acid (4.7%), cyclo(leucyloprolyl) (3.5%), and palmitic acid (3.1%) as minor components. Minor amounts of oleic acid (0.2%) were detected. Our findings showed that the major volatile constituents in the extracts of the mycelium, culture broth, and sclerotium of *L. rhinocerotis* were different. The abundance of fatty acids in the extracts of *L. rhinocerotis* was consistent with a previous report by Lau et al. [2].

### Identification of chemical constituents by UHPLC-ESI-MS/MS

**Figure 3:**
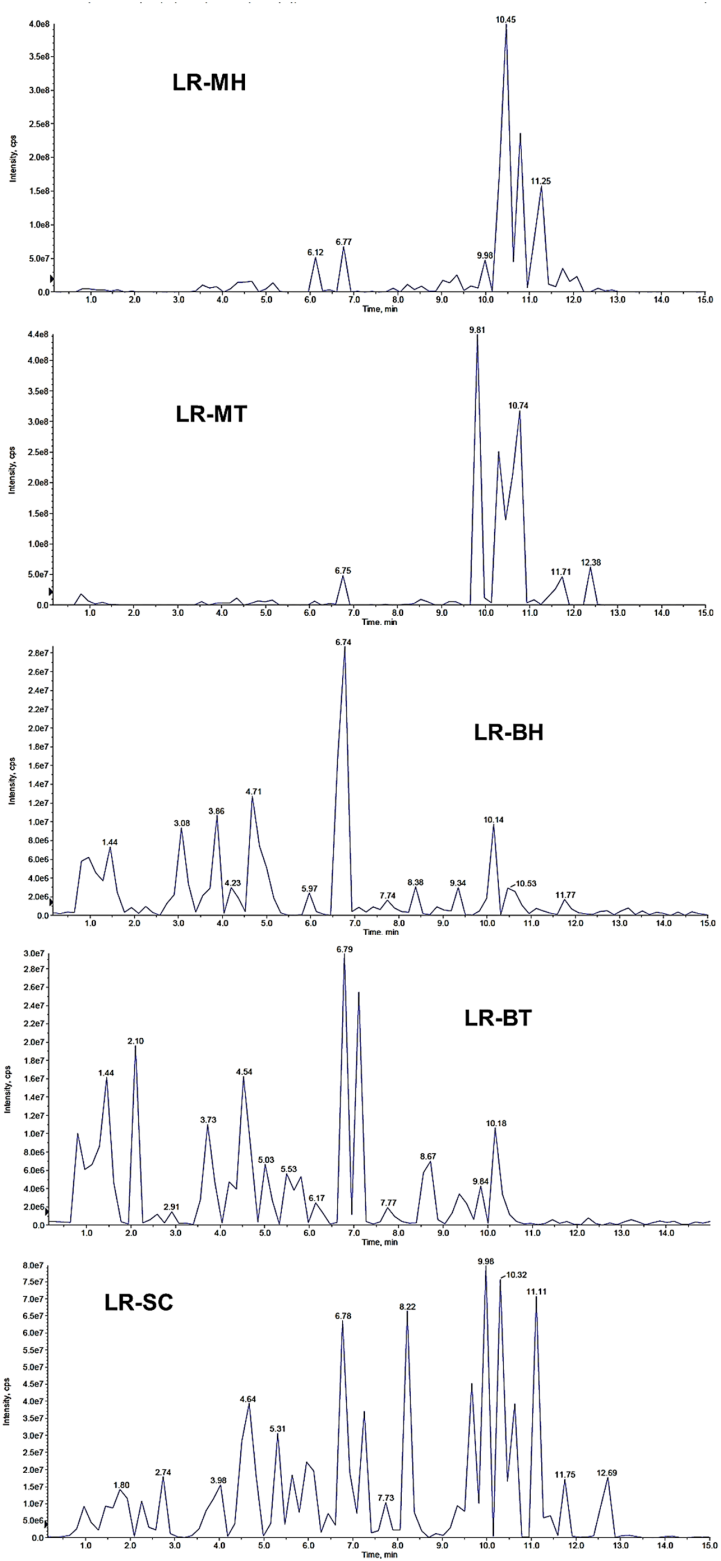
The UHPLC-ESI-MS TIC (negative mode) of the extracts of *Lignosus rhinocerotis*. The profiles of the extracts of the mycelium (LR-MH, LR-MT), culture broth (LR-BH, LR-BT), and sclerotium (LR-SC) were different.

**Figure 4:**
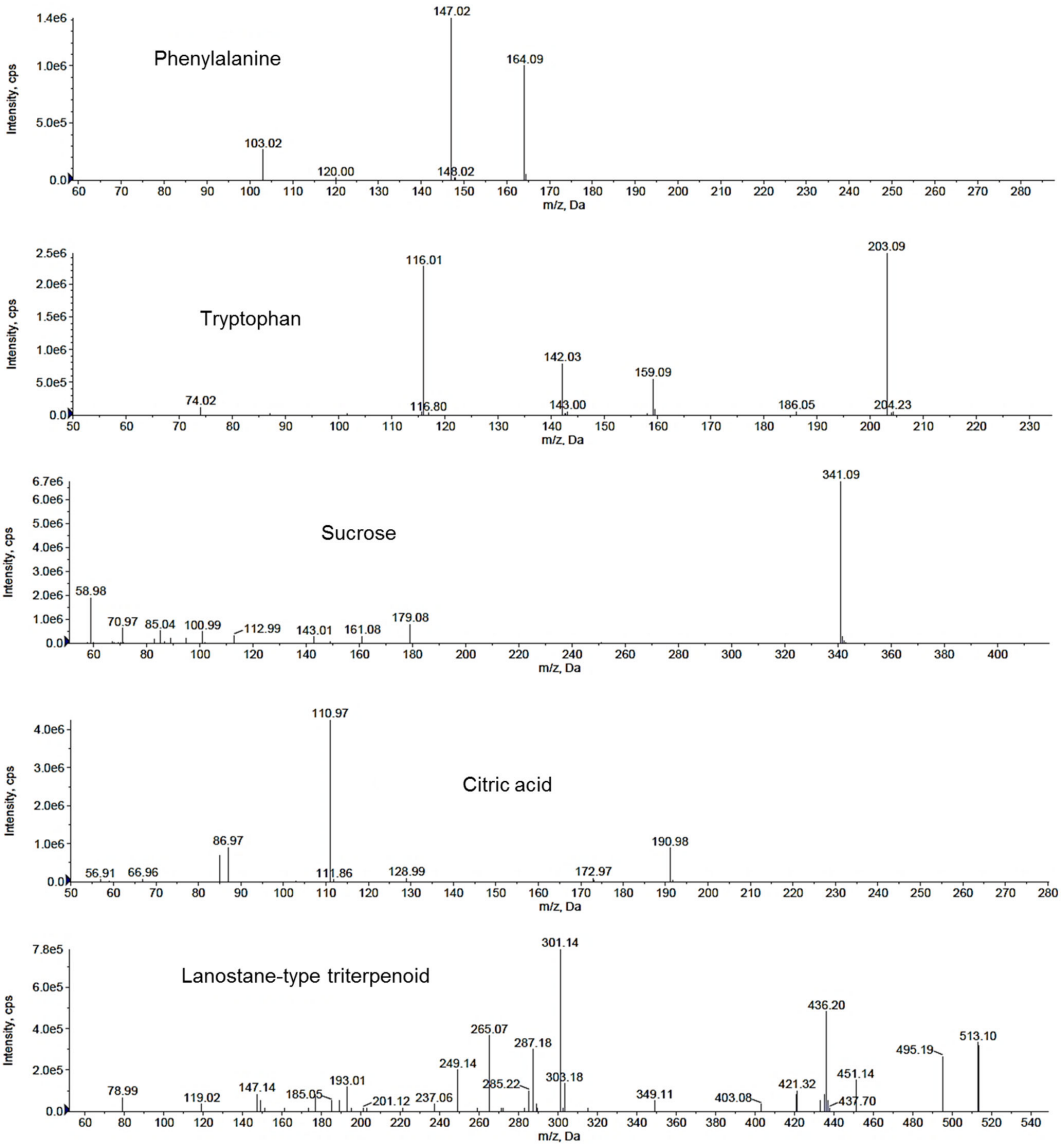
The MS/MS fragmentation (in negative mode) of selected low-molecular-weight compounds in the extracts of *Lignosus rhinocerotis*. Collision energy was set at 35 eV. The compounds were tentatively identified based on their mass fragmentation patterns.

The extracts of *L. rhinocerotis* were also analysed using UHPLC-ESI-MS/MS. The TICs of the extracts are shown in Figure 3. The nature/class of the compounds was determined based on their mass fragmentation patterns (Figure 4) and comparison with literature and databases (e.g., MassBank [http://www.massbank.jp]). Triterpenoids, amino acids, sugars, organic acids, and phenolics were tentatively identified (Tables 7−9). These represent some common metabolites found in most culinary/medicinal mushrooms.

**Table 7:** Chemical constituents in LR-MH and LR-MT based on UHPLC-ESI-MS/MS.

R*_T_* (min)	[M-H]^−^	Massfragments, MS/MS	Suggestedidentification	Reference
**LR-MH**				
1.13	164	147, 120, 103	Phenylalanine	Ying et al. [36] Lu et al. [35] MassBank
1.61	241	197, 167, 141	2-(2-amino-3-imidazol-5-ylpropanoylamino)-3-hydroxypropanoic acid (Histidylserine)	MassBank
3.87	497	451, 433, 333, 225	Lanostane-type triterpenoid	Yang et al. [33] Liu et al. [34]
4.51	451	433, 333, 225	Lanostane-type triterpenoid	Yang et al. [33] Liu et al. [34]
6.12	345	201, 171	Derivative of 9,10-dihydroxy-12Z-octadecenoic acid	MassBank
**LR-MT**				
0.80	341	179, 161, 143, 113,101, 85, 71, 59	Sucrose	Brudzynski and Miotto [37] Taylor et al. [38]
6.11	345	201, 171	Derivative of 9,10-dihydroxy-12Z-octadecenoic acid	MassBank

R*_T_*, retention time.

**Table 8:** Chemical constituents in LR-BH and LR-BT based on UHPLC-ESI-MS/MS.

R*_T_* (min)	[M-H]^−^	Mass fragments, MS/MS	Suggested identification	Reference
**LR-BH**				
0.80	377	341, 221, 179, 161, 97, 87	Hexose-based compound	MassBank
1.29	227	183	Phenolic	-
1.45	241	197, 181, 169, 140	Derivative of emodin	MassBank
4.20	497	451, 433, 225	Lanostane-type triterpenoid	Yang et al. [33] Liu et al. [34]
4.84	451	433, 333, 225, 207, 81	Lanostane-type triterpenoid	Yang et al. [33] Liu et al. [34]
4.99	497	451, 433, 333, 225	Lanostane-type triterpenoid	Yang et al. [33] Liu et al. [34]
**LR-BT**				
1.29	203	159, 143, 116. 74	Tryptophan	Ying et al. [36] MassBank
1.45	241	197, 181, 169, 140	Derivative of emodin	Von Wright et al. [42] MassBank
4.20	497	451, 433, 333, 225	Lanostane-type triterpenoid	Yang et al. [33] Liu et al. [34]
5.01	497	451, 433, 333, 225	Lanostane-type triterpenoid	Yang et al. [33] Liu et al. [34]

R*_T_*, retention time.

**Table 9:** Chemical constituents in LR-SC based on UHPLC-ESI-MS/MS.

R*_T_* (min)	[M-H]^−^	Mass fragments, MS/MS	Suggested identification	Reference
0.96	191	172, 111, 87	Citric acid	John and Shahidi [39]MassBank
3.87	451	433, 333, 225, 207, 143	Lanostane-type triterpenoid	Yang et al. [33] Liu et al. [34]
7.73	513	495, 451, 436, 301, 265, 249, 193	Lanostane-type triterpenoid	Yang et al. [33] Liu et al. [34]
8.22	495	451, 301, 285, 193, 149	Lanostane-type triterpenoid	Yang et al. [33] Liu et al. [34]
10.31	564	504, 279, 224, 153	Lanostane-type triterpenoid	Yang et al. [33] Liu et al. [34]
10.63	504	279, 224, 153	Lanostane-type triterpenoid	Yang et al. [33] Liu et al. [34]

R*_T_*, retention time.

Lanostane-type triterpenoids with high degrees of oxidation have been previously isolated from *Ganoderma* spp. and other polypores including *Inonotus obliquus*, *Wolfiporia cocos*, *Taiwanofungus camphoratus*, and *Laetiporus sulphurous* [32]; hence, their presence in the extracts of *L. rhinocerotis* (a polypore), is not entirely surprising. In negative mode, the triterpenoids were reported to produce two types of molecular ions, i.e., [M-H]^−^ and [2M-H]^−^; fragmentation typically begins with prominent losses of H_2_O or CO_2_ before cleavage takes place on the ring skeleton [33]. A compound (LR-SC, R*_T_* = 7.73 min) produced a deprotonated molecular ion at m/z of 513, and further losses of H_2_O and CO_2_ yielded fragments at m/z 495 and 451, respectively. This fragmentation pattern is similar to ganoderic acid AM_1_, D, and ganoderenic acid B, which can be found in *G. lucidum* [33], [34]. Another compound with an m/z of 497 and fragments at m/z 451 and 433 might possibly have structures similar to ganoderic acid B, D, G, and K, which were reported to form a prominent [M-H-H_2_O]^−^ ion at m/z 497. Other compounds with an m/z of 495 and fragments at m/z 451, 301, and 193 in the extracts were also suspected to be lanostane-type triterpenoids since they possessed fragments considered to be characteristics of ganoderic acids.

Two amino acids having hydrophobic side chains were identified from the extracts. Their mass fragmentation patterns were in agreement with previous reports [35], [36]. Phenylalanine (LR-MH, R*_T_* = 1.13 min) exhibited a deprotonated molecular ion ([M-H]^−^) at m/z 164 and a mass fragment at m/z 147, possibly corresponding to the further loss of an amino group (−NH_2_). Tryptophan (LR-BT, R*_T_* = 1.29 min) gave a deprotonated molecular ion at m/z 203. Further loss of a carboxyl group (CO_2_) produced a fragment at m/z 159. Identification of free amino acids in the extracts of *L. rhinocerotis* corroborates previous findings on the presence of amino acids in the aqueous alcohol extract of mushrooms [24]. Hexoses (6-C sugars) are characterised by m/z fragments at 179, 161, 143, 113, and 89 [37]. A compound (LR-MT, R*_T_* = 0.80 min) with an m/z of 341 was determined to be sucrose based on postulated cleavage of the glycosidic bond to produce fragments at m/z 179 and 161 [37], [38]. Another compound (LR-BH, R*_T_* = 0.80 min, LR-MH) with an m/z of 341 had a constant loss of 162 units, consistent with the loss of a hexose moiety to produce a fragment at m/z 179.

Previous studies have revealed that mushrooms are rich in phenolic compounds [24], [27]. In this study, however, very few phenolics were identified in the extracts (data not shown), and this corroborated the low phenolic content (Table 3). A compound in LR-SC (R*_T_* = 0.96 min) with m/z 199 and mass fragment at m/z 111 was tentatively identified as citric acid, in accordance with the literature [39]. Organic acids are commonly found in mushroom fruiting bodies [40]. Citric acid is an important intermediate in the Krebs cycle, which is one of the major cellular energy-yielding pathways. The lack of organic acids in the mycelium and culture broth might be because that these were used to support rapid vegetative growth in the mycelia, as proposed by Pinto et al. [41]. A compound present in LR-BH and LR-BT (R*_T_* = 1.45) produced a [M-H]^−^ ion at m/z 241 and fragments at 197, 181, 169, and 140. Its fragmentation pattern closely resembled that of 1,3,8-trihydroxy-6-methylanthraquinone (emodin), which was previously reported to be present in a wild mushroom (*Dermocybe sanguinea*) [42]. The compound was deduced to be a type of anthraquinone based on the similarity of fragmentation patterns.

**Figure 5:**
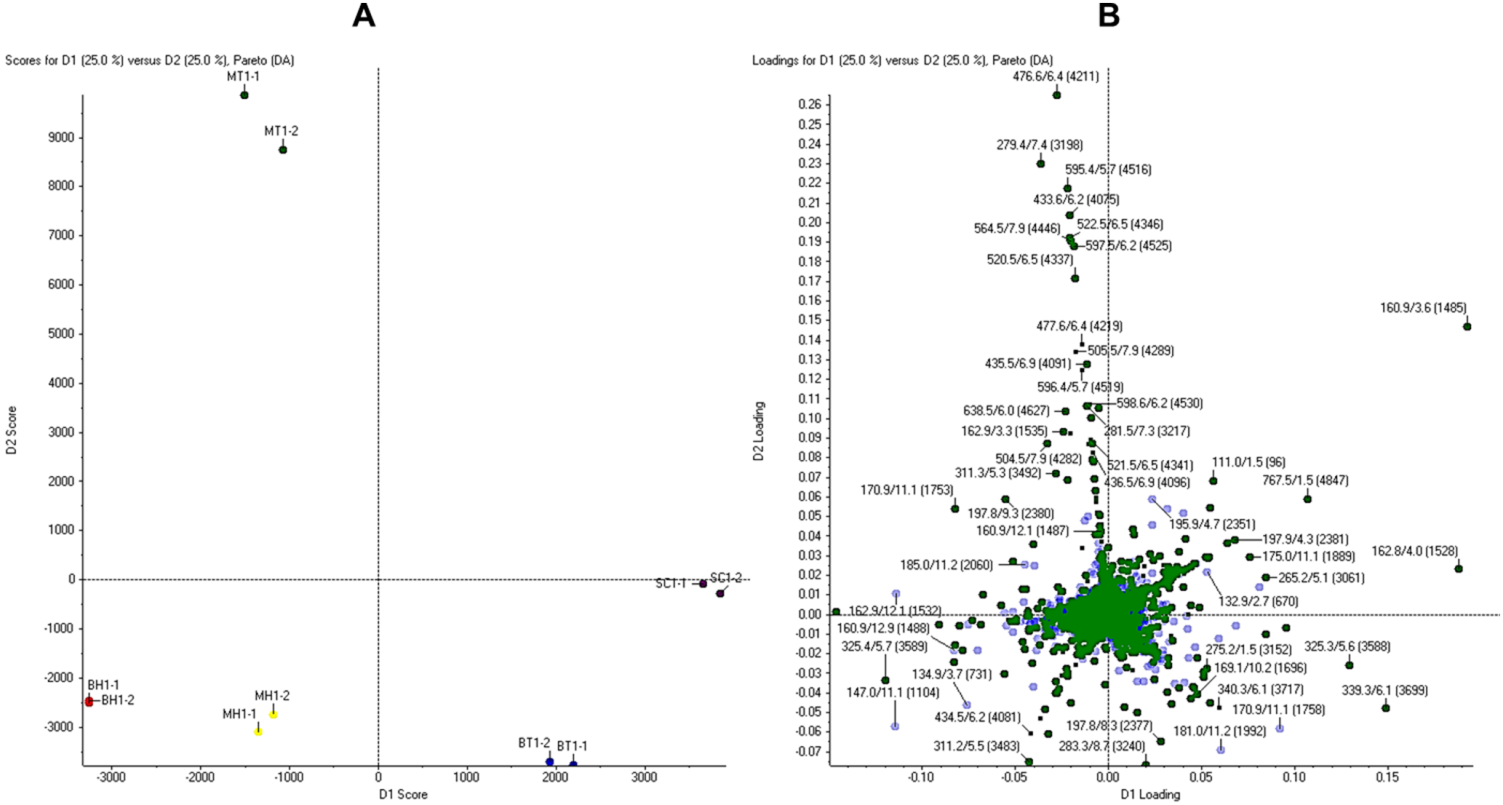
The UHPLC-ESI-MS (m/z 100–1000) principal component analysis of the extracts of *Lignosus rhinocerotis*. Duplicate analysis of the extracts of mycelium from shaken (MH1, MH2) and static (MT1, MT2) conditions, culture broth from shaken (BH1, BH2) and static (BT1, BT2) conditions, and sclerotium (SC1, SC2) were performed. (A) Score plot revealed that mycelia from shaken and static conditions were distinct from the sclerotium. (B) Loading plot with multiple ions common to all extracts (centre) and marker ions far from the centre, e.g. m/z 161, 325, 339, and 766, were characteristic of individual extracts. The identification of the compounds is warranted for determining biomarkers for *L. rhinocerotis* from different morphological/developmental stages.

Mass signals from the UHPLC-ESI-MS/MS for LR-MH, LR-MT, LR-BH, LR-BT, and LR-SC were subjected to PCA. In the score plot (Figure 5A), LR-SC and LR-BT (positive region) were separated from LR-MH, LR-BH, and LR-MT (negative region) by the first principal component. The LR-MT (positive region) could be distinguished from LR-MH and LR-BH (negative regions) by the second principal component. Extracts from shaken cultures (LR-MH and LR-BH) were clustered together. Some of the compounds in the mycelia might have been secreted into the culture broth, and hence, at the harvest time (day 15), the chemical profile of the intracellular (LR-MH) and extracellular (LR-BH) constituents were comparable. The results also showed that chemical constituents in the mycelium under shaken and static conditions were distinct from those of the sclerotium. A loading plot (Figure 5B) was generated to identify the variables that contributed to the differences in the extracts. It was found that several marker ions are far from the centre of the loading plot, suggesting that the concentrations of these compounds in the extracts were highly varied.

### Bioactivities in relation to chemical constituents

The considerable variation in the chemical profiles, as described above, might be the main reason for differences in the antioxidant capacities between the mycelium, culture broth, and sclerotium of *L. rhinocerotis*. Mushroom extracts are good sources of phenolic compounds, and the correlation between phenolics and antioxidant capacity implies the possible roles of these compounds as antioxidants [43]. Due to the low phenolic content in the extracts of *L. rhinocerotis*, the roles of other compounds present in the extracts and have been reported to exhibit antioxidant capacities, such as triterpenoids [44], organic acids [24], proteins [45], ergosterol, sterol derivatives, and fatty acids [46], should be considered. As suggested by Carvajal et al. [24], synergistic effects of the antioxidant compounds in the extracts should not be ruled out.

Extensive work has been done to identify low-molecular-weight cytotoxic compounds from medicinal mushrooms and their possible modes of action [47]. The cytotoxic and apoptotic effects of triterpenoids, such as ganoderic acids from *G. lucidum* [32], [34] and inotodiol from the sclerotium of *I. obliquus* [48], have been documented. Other classes of potentially cytotoxic metabolites are fatty acids, their conjugated forms, and sterols. The most abundant fatty acid in the extracts of *L. rhinocerotis* was linoleic acid, followed by palmitic and steric acids. Previously, it was reported that linoleic acid did not exert growth inhibition against the testosterone-dependent MCF-7aro cell [49]. Palmitic acid, on the other hand, has been shown to induce apoptosis in human leukemic cells (MOLT-4) [50], and stearic acid was reported to inhibit colony-forming abilities of human cancer cells [51].

### The potential of mycelium and culture broth as a substitute for sclerotium

The aqueous methanol extracts, composed of low-molecular-weight compounds, of the mycelium and culture broth of *L. rhinocerotis* showed comparable bioactivities to the sclerotium. In the antioxidant capacity assays, LR-BT was the most potent extract with respect to its ABTS radical scavenging activity, ferric and cupric ion reducing capacities, ferrous ion chelating potential, and inhibitory effect on lipid peroxidation. This indicated that, in terms of antioxidant capacity (Table 1), the sclerotium is not superior compared to the mycelium and culture broth. Secondly, results from the MTT assay showed that all extracts were non-cytotoxic (IC_50_>200 µg/ml) against a panel of mammalian cell lines. This implied that *L. rhinocerotis* from different morphological/developmental stages (i.e., mycelium and sclerotium) do not contain low-molecular-weight, cytotoxic compounds in abundance. It should be noted that in this study, an exhaustive extraction using aqueous methanol was employed; hence, the resulting extracts would contain lower proportions of non-polar constituents than extracts prepared from other solvents, such as hexane, chloroform, dichloromethane and/or ethyl acetate. A more detailed investigation (e.g., successive extraction using solvents of increasing polarity and/or fractionation of the aqueous methanol extracts) is warranted should bio-prospecting of cytotoxic metabolites from *L. rhinocerotis* be desired.

According to Lau et al. [2], the proximate composition and some nutritional attributes of the mycelium were comparable to those of the sclerotium. This has provided a basis for considering the mycelium an alternative to the sclerotium. The extensive chemical profiling by GC-MS, UHPLC-ESI-MS/MS, SDS-PAGE, and SELDI-TOF-MS in this investigation provided insight into the nature of different low-molecular-weight compounds in *L. rhinocerotis*; nevertheless, further confirmation of these compounds would require additional chemical investigation which is currently in progress. Previously, we found that protein profiles of *L. rhinocerotis* cultured in a stirred tank reactor and static cultures were different [9]. Our results here demonstrated that culture conditions also affected the composition of low-molecular-weight compounds and their bioactivities. The strong antioxidant capacity of LR-BT and cytotoxicity of LR-MT against MCF 10A might be due to compounds produced specifically during static conditions. The chemical basis for this observation has yet to be elucidated, but the lack of aeration and spatial homogeneity as well as the merging of growth phases in static cultures might have effects on the biosynthesis of secondary metabolites [9]. In fact, it has been reported that production of microbial secondary metabolites is enhanced in stressed conditions. For instance, oxygen limitation has been shown to enhance the production of ganoderic acid by *G. lucidum* in submerged cultures [52]. Several workers have investigated the correlation between culture conditions and bioactivities [14], [25]. The effects of culture conditions on the production of chemical constituents and their bioactivities require further investigation.

Mycelium, culture broth, and sclerotium represent mushroom samples from different cultivation techniques. Some of the advantages of liquid fermentation over solid-substrate fermentation, such as shorter time, greater quality control, and lesser contamination, might favour large-scale production of mycelium and culture broth as substitutes for either the cultivated or wild sclerotia for use in formulation of nutraceuticals. The diversity in the chemical constituents between mycelium, culture broth, and sclerotium, as demonstrated by the chromatographic and mass-spectrometric analyses, warrants future work pertaining to the metabolomics of mushrooms from different morphological/developmental stages and culture conditions. Regarding our results from bioactivity evaluation and chemical profiling, *L. rhinocerotis* from liquid fermentation merits further consideration as a source of functional ingredients.

## Supporting Information

Figure S1 GC-MS TIC of LR-MH.
(TIF)

Figure S2 GC-MS TIC of LR-MT.
(TIF)

Figure S3 GC-MS TIC of LR-BH.
(TIF)

Figure S4 GC-MS TIC of LR-BT.
(TIF)

Figure S5 GC-MS TIC of LR-SC.
(TIF)
