# Supplementary figures and images for: The Potential of Mycelium and Culture Broth of Lignosus rhinocerotis as Substitutes for the Naturally Occurring Sclerotium with Regard to Antioxidant Capacity, Cytotoxic Effect, and Low-Molecular-Weight Chemical Constituents
Source: PLoS One. 2014 Jul 23;9(7):e102509. doi: 10.1371/journal.pone.0102509 (PMC4108328; doi:10.1371/journal.pone.0102509)

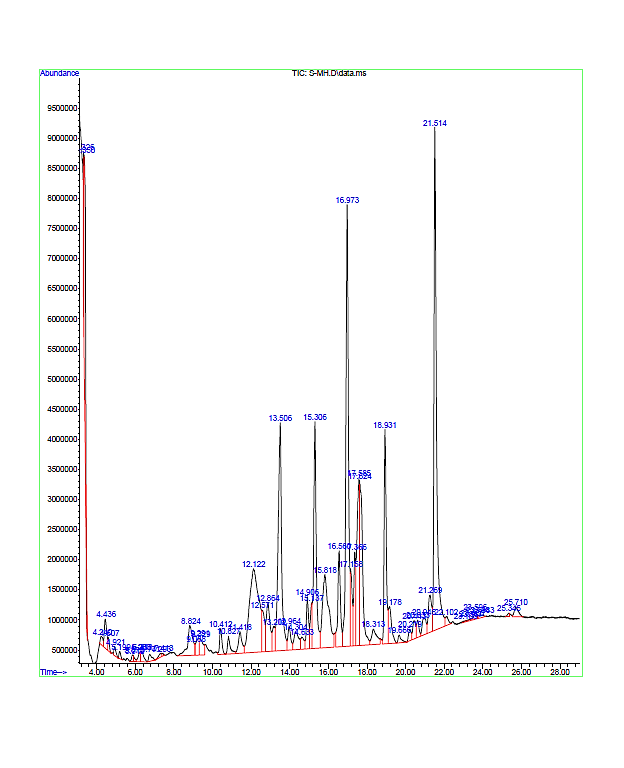

Supplement: Figure S1 — GC-MS TIC of LR-MH. (TIF) [file pone.0102509.s001.tif]

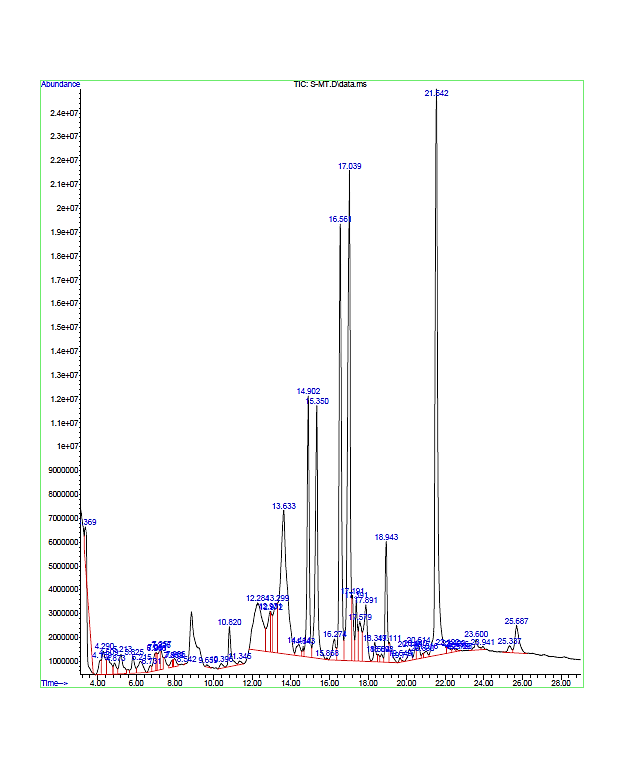

Supplement: Figure S2 — GC-MS TIC of LR-MT. (TIF) [file pone.0102509.s002.tif]

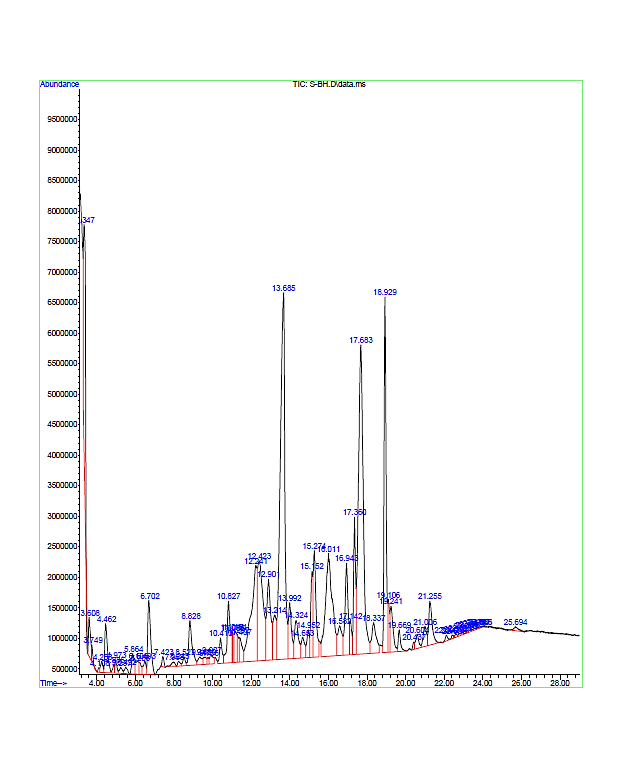

Supplement: Figure S3 — GC-MS TIC of LR-BH. (TIF) [file pone.0102509.s003.tif]

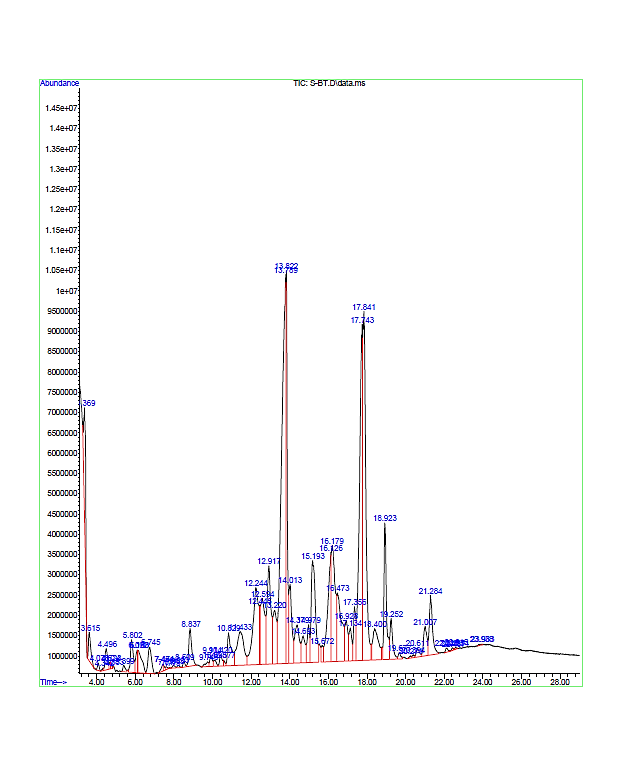

Supplement: Figure S4 — GC-MS TIC of LR-BT. (TIF) [file pone.0102509.s004.tif]

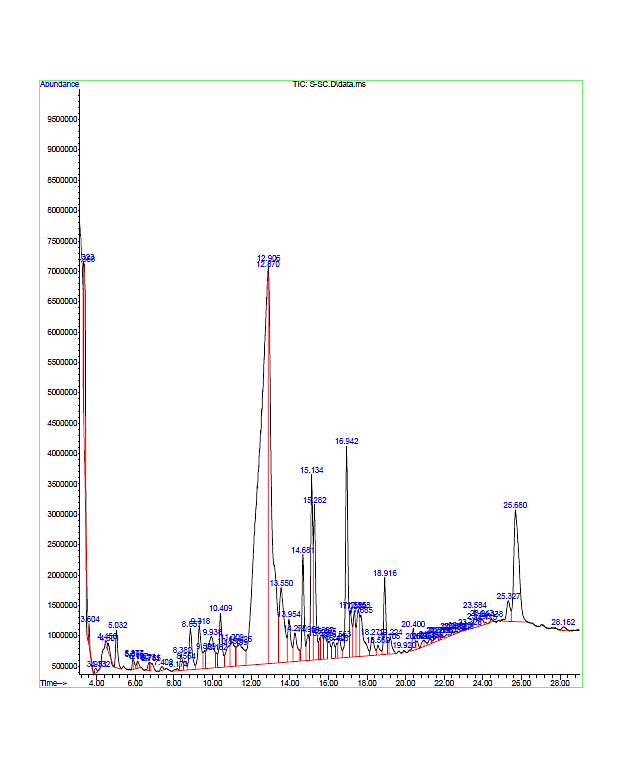

Supplement: Figure S5 — GC-MS TIC of LR-SC. (TIF) [file pone.0102509.s005.tif]
